# Draft Genome Sequence of *Cryptococcus flavescens* Strain OH182.9_3C, a Biocontrol Agent against Fusarium Head Blight of Wheat

**DOI:** 10.1128/genomeA.00762-13

**Published:** 2013-09-26

**Authors:** Xiaoqing Rong, Brian B. McSpadden Gardener

**Affiliations:** Department of Plant Pathology, the Ohio State University-Ohio Agriculture Research and Development Center (OARDC), Wooster, Ohio, USA

## Abstract

*Cryptococcus flavescens* strain OH182.9_3C (3C) is a novel biopesticidal agent that can be used to control fusarium head blight of wheat. Here we present the draft genome sequence for 3C, the first for the species *C. flavescens*. Additionally, several genes that may contribute to the biocontrol activities of 3C were identified *in silico.*

## GENOME ANNOUNCEMENT

Fusarium head blight (FHB) is predominantly caused by *Fusarium graminearum* Schwabe (teleomorph: *Gibberella zeae* [Schwein.]) (1) and results in up to billions of dollar losses per epidemic year in the United States (2–4). *Cryptococcus flavescens* strain OH182.9 (NRRL Y-30216) is a basidiomycete yeast isolated from wheat anthers collected from fields in the United States that had experienced epidemics of FHB (5). Applications of OH182.9 during anthesis, when the wheat heads are most susceptible to *F. graminearum* infection, were previously shown to significantly reduce FHB damage in green house and field experiments (5–7). OH182.9_3C (3C, NRRL Y-50378) is a fungicide-resistant variant of OH182.9 that can be combined with certain fungicides to further enhance control of FHB on wheat and barley.

A 300-bp fraction of randomly sheared 3C genomic DNA was sequenced on an Illumina Genome Analyzer II (Illumina, San Diego, CA) for 76 cycles. The process generated 46,300,240 paired-end reads. The quality-trimmed reads (Sanger *Q*, ≥20, 95.72% of total reads) were assembled using Velvet v 1.0 (8, 9) with a hash length (k-mer) of 31 nucleotides (nt) and a coverage cutoff of 5 nt. The resulting assembly had 22,830,355 nt in 3,580 scaffolds with an *N*_50_ of 71,386 nt and a maximum scaffold length of 374,074 nt. Short scaffolds were removed from the assembly by use of a threshold of minimum length of 300 nt, leaving 712 scaffolds. The final assembly had a total of 22.79 million nucleotides and a median coverage of 45×.

*Ab initio* gene prediction was performed on the assembly by Augustus 2.6.1 (10) using *Cryptococcus neoformans* var. *neoformans* JEC21 as the model. A total of 9,063 open reading frames (ORFs) with a mean length of 1,802 bp were predicted on 589 out of 712 scaffolds. Gene functions were explored by a BLASTx search querying the whole assembly against the NCBI nonredundant protein sequences (nr) using an *E* value cutoff of 10^-100^. A search for genes related to biological control was conducted. Chitinases and 1,3-β-glucanases produced and secreted by biocontrol agents may suppress fungal pathogens by degrading their cell walls and/or by inducing host resistance (11). BLASTx hits of multiple glycosyl hydrolases were predicted to have secretion signals by use of SignalP v 4.1 (12). Among the predicted proteins, one was 72% identical to chitinases, one 53 to 57% identical to glucan 1,3-β-glucosidases, and two more were 59 to 75% identical to endo-1,3(4)-β-glucanases in *C. neoformans* and *C. gattii*. Nonribosomal peptide synthases were previously implicated in the biosynthesis of antimicrobial peptibols that have biocontrol activities in *Trichoderma* spp. (13). Putative nonribosomal peptide synthases were found in several 3C scaffolds. Interestingly, the top hits for two of those genes were nonribosomal peptide megasynthases (NRPS) from ascomycetes and bacteria, not *Cryptococcus* or other basidiomycetes. Further investigation into these genes through expression analysis and bioassays is warranted.

### Nucleotide sequence accession numbers.

This whole-genome shotgun project has been deposited at DDBJ/EMBL/GenBank under the accession number CAUG00000000. The version described in this paper is the first version, CAUG00000000.1.
